# Construction of exchange integrated information chain management model leading by information nurse for large instrument and equipment in operating room

**DOI:** 10.1186/s12911-021-01417-w

**Published:** 2021-02-10

**Authors:** Jinghua Dai, Xiaoqiang Ren, Peng Wu, Xiangdong Wang, Jiang Li, Haiyan Bian, Lingai Ren, Chunmei Wu

**Affiliations:** 1 Department of Anesthesia Surgery, Shanxi Provincial People’s Hospital, No. 29 of Twin Towers Street, Yingze District, Taiyuan, 030012 China; 2 Department of Information Office, Shanxi Provincial People’s Hospital, Taiyuan, 030012 China; 3 Department of Material Supply Department, Shanxi Provincial People’s Hospital, Taiyuan, 030012 China

**Keywords:** Operating room, Information nurse, Equipment management, Chain flow, Large instruments and equipment

## Abstract

**Background:**

This study aims to explore the information chain management model of large instrument and equipment inter-working in the operating room (OR) led by information nurses.

**Methods:**

Through the chain management process of large instruments and equipment in the OR, which was based on information nurses, the management model of inter-working and integrating information chain was established, the key links were controlled, and the whole life cycle management of instruments and equipment from expected procurement to scrapping treatment was realized. Using the cluster sampling method, 1562 surgical patients were selected. Among these patients, 749 patients were assigned to the control group before the running mode, and 813 patients were assigned to the observation group after the running mode. The related indexes for large instrument and equipment management in the department before and after the running mode were compared.

**Results:**

In the observation group, the average time of equipment registration was (22.05 ± 2.36), the cost was reduced by 2220 yuan/year, and the satisfaction rate of the nursing staff was 97.62%. These were significantly better, when compared to the control group (*P* < 0.05). Furthermore, the awareness rate of the whole staff for equipment repair application was 95.12%, and the arrival time of maintenance personnel and the examination and approval time of equipment management were greatly shortened (*P* < 0.05).

**Conclusion:**

The integrated management model of large instrument and equipment interworking in the OR based on chain flow realizes the whole life cycle management of instruments and equipment, which is essential to improve management efficiency.

## Background

As the most comprehensive department of multi-disciplinary, the OR continues to face many challenges brought by the increase in instruments. Ensuring the efficient and normal running of these medical devices is an important link to the success of the surgery and safety of the patients [1]. Meanwhile, higher requirements are proposed for the professional skills and instrument management (IM) of OR nurses [2]. Large instruments in the OR need to undergo a series of multi-department comprehensive management measures, from expected procurement to final disposal [3]. Therefore, the management of large-scale instruments is a chain type that needs the cooperation of multiple departments, and its chain flow structure is very obvious [4]. Chain process management refers to management activities that maintain the effective continuity of each link [5]. At present, the informative construction of international hospitals at all levels has achieved remarkable progression [6, 7]. Research is committed to medical equipment manufacturers more system interoperability between the network construction, such as OR.NET project [8], the United States MDPnP project [9] or Japan fabrics project [10]. These could achieve connectivity on the management of instruments and equipment between departments, but to a certain extent, the economic cost is higher. How to make the social value greater than the economic cost is still a problem to be resolved. Based on the integrity of information transmission, convenience of operation, accuracy of screening and other characteristics of the information system, hospitals independently design the large-scale IM system for the OR, with the information nurse having the leading role, and combs the process nodes involved in three management levels: hospital—use department—maintenance department. This is correlated to the whole life cycle of the IM flow, and standardizes business content at all levels. Through this, and with the help of information system intercommunication and integration, a multi-level closed-loop management model was formed to improve the management efficiency of large-scale instruments.

## Data

In July 2019, the data of Neurosurgery, Orthopedics, General Surgery, Cardiothoracic Surgery and Gynecology of a third-class hospital in Taiyuan in the operating the management model were collected and evaluated. By cluster sampling, 749 patients were selected for the control group before the use of the inter-working and integrating information chain management model, and 813 patients from these departments were selected for the observation group after the implementation of the model. Inclusion criteria: the length of hospitalization stay was more than 24 h; one or more instruments were needed during the surgery. Exclusion criteria: after the patient entered the OR, the surgery was not carried out for some reason, or the instrument was not used during the surgery; the types of instruments used during the surgery did not conform to the category of large-scale instruments [11, 12].

## Methods

### Establishment of an interdisciplinary team and a new model of information research and development (R&D)

A three-party R&D team that consisted of engineers of the information department, information nurses of the OR, and administrators of the material supply department was established. This team included the following: Two information nurses in the OR have a master’s degree. These nurses have attended the basic training class for nursing information ability conducted by Professor Zhang Bolun at Taiwan National Yangming University, which is a three-year "Million Planned Talent Introduction Project", and obtained the TIGER international information nurse certification. Two key engineers in the information department have a master degree in computer science, and have been engaged in clinical information work for more than 10 years. The administrator of the material supply department was responsible for software operation and management, and was familiar with large-scale IM processes.

The new model of information research and development is a three-way linkage team led by information nurses, which comprised of the administrator of the material supply department, information nurses and engineers in the hospital. Among them, the administrator of the material supply department corresponds to the information needs provided for clinical work, while information nurses corresponds to innovation, improvement and design based on clinical work and basic knowledge of information. Engineers and information nurses closely work together to independently develop information systems.

### The system flowchart

**Fig. 1:**
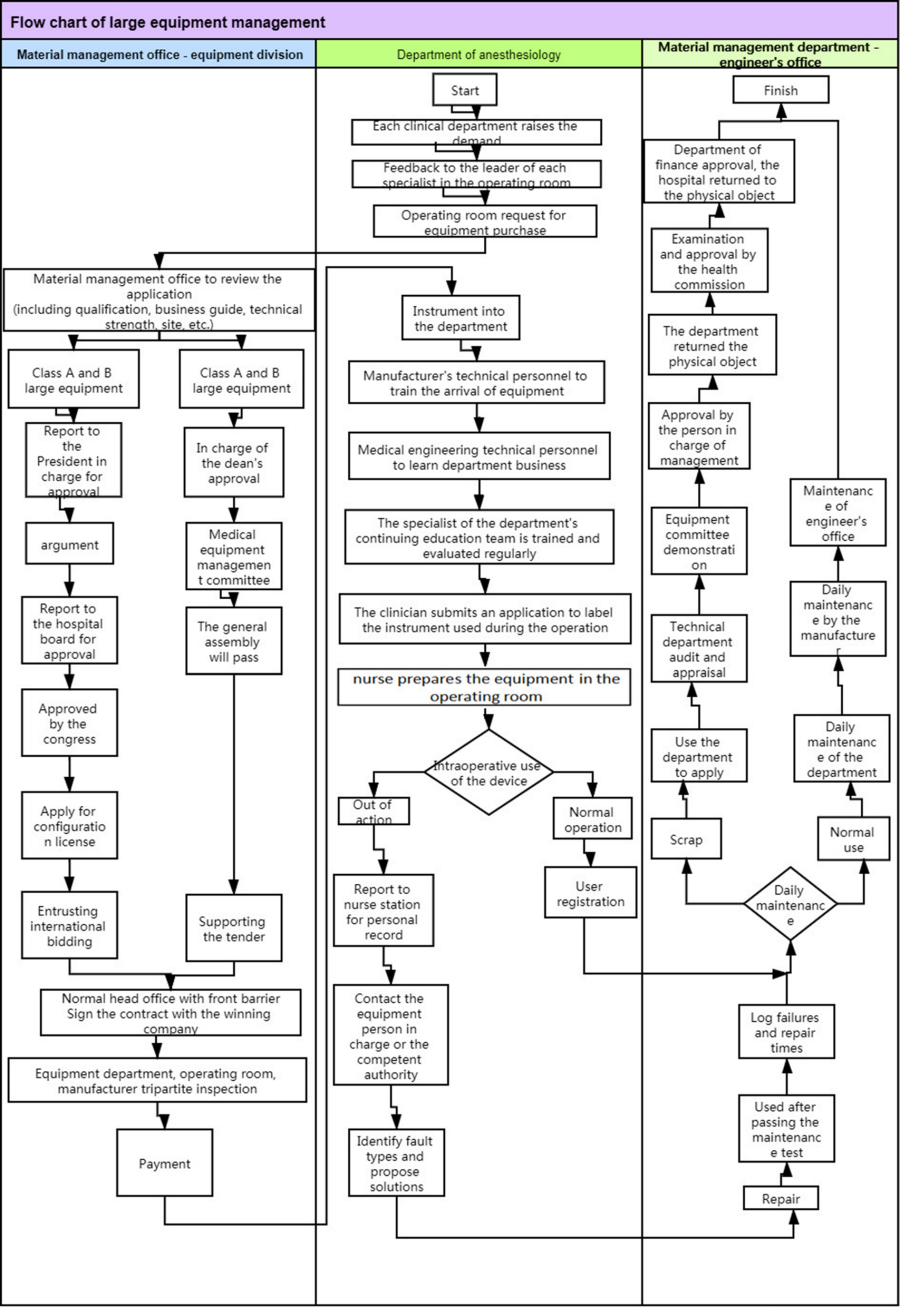
system flow chart

The R&D team combed the process nodes, and drew the cross-functional flow chart for the large-scale IM in the OR (Fig. 1), according to the Detailed Rules for the Implementation of the Evaluation Standard of Shanxi Three—level General Hospital (revised in 2012) and clinical practice. The IM chain involves the following: hospital management level (equipment department), use department management level (OR), and maintenance management level (engineer room).

The information management model in the control group includes the information management platform of medical equipment archives, the automatic office (OA) system, and the maintenance of management system (MS) of the material management office.

### Construction and implementation of the inter-working and integrating information chain management model

#### Design and implementation of cross-department flow chain of instruments and equipment

**Fig. 2:**
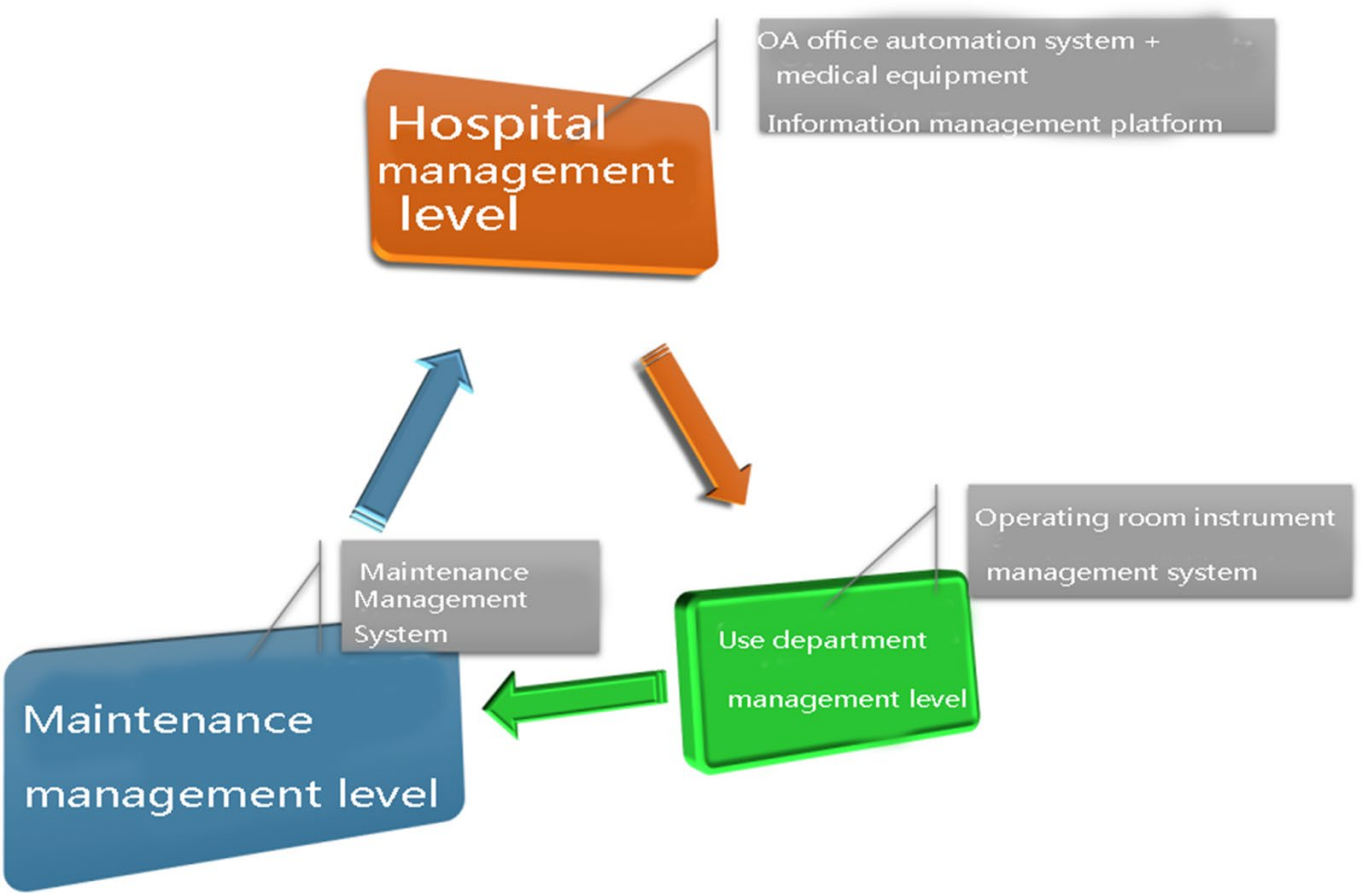
multi-layer closed-loop management mode of equipment

**Fig. 3:**
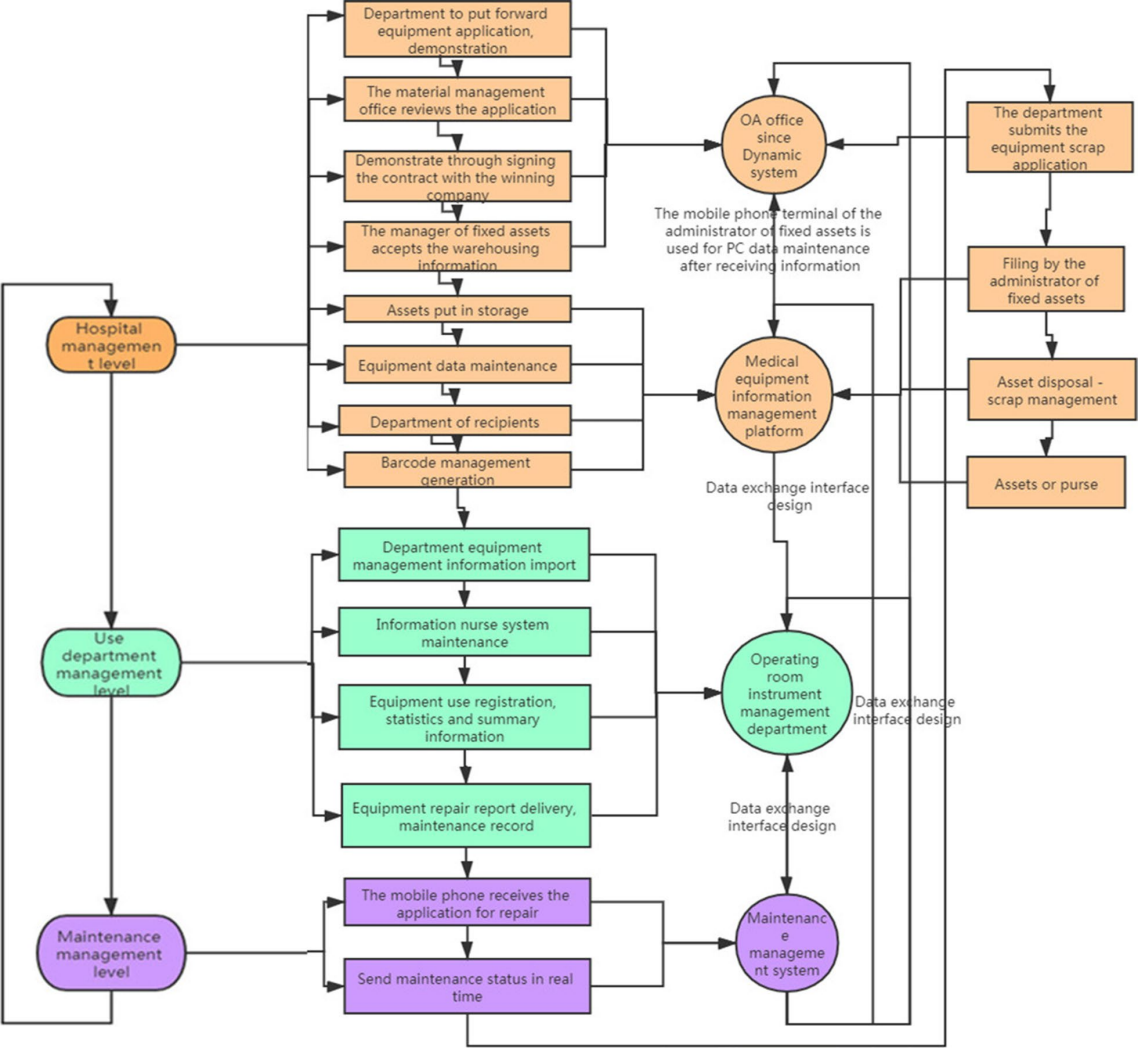
information chain management content at all levels

With the use of the characteristics of information system transmission integrity, the registration content involved in the equipment management chain (Fig. 2) is linked and combined (Fig. 3). The data interface design realizes the purpose of the inter-working and integrating information. The software system involved in the hospital management level includes the OA and the medical equipment information management platform. The department use management level involves the OR-IM system (self-designed). The maintenance management level involves the maintenance MS.

#### Design and implementation of large instrument nursing business chain in OR led by information nurses

##### 1. Preparation before system development

Firstly, the information nurse set up the large instruments of operating room (OR) and equipment management database, including the equipment name, instrument registration information, statistical analysis content, equipment maintenance and personnel information. Secondly, according to the flow chart of cross-functional departments, the information nurse analyzed the business chain of instrument and equipment nursing management. Focusing on the following aspects: how to structure the content so as to achieve the standardization of information system window content design; how to query data according to conditions and extract data automatically; how to realize the real-time update of the instrument usage status and design the interface of data exchange for maintenance barrier reservation. The information nurse used Microsoft Excel 2010 and its internal Excel VBA tool to write programs and to provided preliminary form design schemes and communicate system functions with engineers by means of dynamic demonstration.

##### 2. System development and test optimization

The information engineers compiled the software program according to the requirements and prototype of design provided by the information nurses. According to the actual requirements of instrument chain management, data exchange interface for maintenance and fault reporting was designed and reserved in order to realize the integration of system and hospital maintenance system. After the software was implemented, the information nurses would conduct a comprehensive joint debugging test on the system and submit the system test report to the engineers after finding any problems. The engineers would further modify the system according to the test reports.

### Effect evaluation

Indicators correlated to large-scale IM in the department: The researcher himself tested the average registration time of the instrument used of these two groups of patients. The calculation method was as follows: the time of manual record was calculated from the first word of the record to the completion of the record, the recording time of the information system was calculated from the input of patient information to the completion of the input, and the statistics for the registration qualified rate was conducted. The cost and benefit of these two management models were calculated, which mainly reflected in paper savings. The satisfaction rate of these two IM models was evaluated, the Likert five—levels method was used, and 5 points to 1 point were assigned from very satisfied, general satisfied, satisfied, dissatisfied and very dissatisfied. Investigation subjects for the above-mentioned indicators were all front-line nurses.

Indicators related to cross-departmental IM: For the awareness rate of the whole staff for equipment repair application, the arrival time of maintenance personnel (the total time from sending the service information to the maintenance personnel arriving at the site for maintenance), the approval time of the equipment scrap, the approval time of the equipment purchase, and the approval time of purchase of relevant components of these equipment, all data were obtained from the information platform after the implementation of the inter-working and integrating information chain management model, while data before the implementation were obtained from the paper record text.

### Statistical analysis

The data were statistically analyzed using statistical software SPSS 16.0, with t-test, rank sum test and Chi-square test. Measurement data were expressed as mean ± standard deviation (x ± SD). Nonparametric data were expressed in quartile M (P25, p75). The data were tested for normality: non-normally distributed data were compared using non-parametric Wilcoxon rank sum test, while normally distributed data were compared using t-test. Count data were expressed in percentage (%), and evaluated using Chi-square test. *P* < 0.05 was considered statistically significant, which indicated that the two groups had different statistical values and the intervention measures were effective.

## Results

### Comparison of the general situations of the two groups

There were no significant differences between the two groups in terms of age, gender, department distribution, and the number of instruments used during the surgery (*P* > 0.05). Hence, these two groups were comparable (Table 1).

**Table 1:** Comparison of general conditions between the two groups

Groups	N	Age (years, x ± s)	Gender		The operation time (h, x ± s)	Department distribution					Number of instruments used during operation			
			M	F		Neuro	Orthopedics	Cardiothoracic	General Sugery	Gynecology	1	2	3	> 3
Observation group	813	52.83 ± 10.15	356	457	4.33 ± 1.64	159	198	123	160	173	80	300	347	86
Control group	749	52.03 ± 9.11	350	399	4.28 ± 1.35	147	183	118	142	159	74	285	314	76
*t/z/x*^*2*^	–	1.63	1.36		0.66	0.21					0.26			
*p*	–	0.10	0.43		0.68	0.67					0.64			

### Indicators correlated to large-scale IM in the departments

There were significant differences between these two groups in terms of average registration time and the registration qualified rate of the instrument used during the operation. The registration time was significantly shortened and the registration qualified rate significantly increased in the observation group (*P* < 0.01, Table 2).

**Table 2:** Comparison of the average time required for intraoperative instrument registration and the qualified rate between the two groups

Groups	N	Registration time (s)	Instrument registration qualification rate (%)
Control group	813	54.09 ± 4.99	80.93 (658/813)
Observation group	749	22.05 ± 2.36	98.00 (734/749)
***t/x***^***2***^	–	28.16	117.02
***p***	–	0.000	0.000

The survey of the satisfaction rate of nursing staff on these two IM models was showed in Table 3. The satisfaction rate was 54.76% in the control group, and 97.62% in the observation group.

**Table 3:** Satisfaction survey of the management mode of the two instruments and equipment

Groups	N	Nursing staff satisfaction					*x*^*2*^	*p*
		Great satisfaction	General satisfaction	Satisfaction	Dissatisfaction	Very Dissatisfied		
Control group	42	0	11	12	13	6	63.17	0.000
Observation group	42	34	7	0	1	0		

### Indicators correlated to cross departmental IM

Analysis of indicators correlated to cross-departmental information transmission speed: There were 63 items of maintenance information in the control group, and 41 items of maintenance information in the observation group. The information transfer efficiency was compared between these two models. The results revealed that the awareness rate of the whole staff for equipment repair application and arrival time of maintenance personnel significantly improved in the observation group, and the differences were statistically significant (*P* < 0.01, Table 4).

**Table 4:** Comparison of information transfer speed between two groups of management modes across departments

Groups	N	The awareness rate of all staff in the equipment repair report (%)	Maintenance crew arrival time (h)
Control group	63	11.11 (7/63)	24.31 ± 3.01
Observation group	41	95.12 (39/41)	18.24 ± 1.87
***t/x***^***2***^	–	71.06	15.02
***p***	–	0.000	0.000

Analysis of instrument-related approval time: the examination and approval time was significantly shortened in the observation group, and the difference between these two groups was statistically significant (*P* < 0.01, Table 5).

**Table 5:** Comparison of equipment approval time of the two management modes

Groups	Equipment scrap approval time (days)	Equipment purchase approval time (days)	Equipment related components purchase approval time (days)
Control group	10.34 ± 1.56	18.09 ± 1.44	12.49 ± 2.40
Observation group	4.83 ± 1.57	9.71 ± 0.88	4.12 ± 0.61
***t***	17.77	33.52	21.85
***p***	0.000	0.000	0.000

## Discussion

Luping Li, Vice-President of China Hospital Association, first released "The Patient Safety Objectives of China Hospital Association (2019 version)" at the 2019 China Hospital Quality Conference [13], emphasizing the importance of the safe use of medical equipment. At present, the development model of the hospital information system was basically to purchase company software, which cannot fully meet the work needs [14, 15]. Nursing staff have limited information ability, and needs to rely on the assistance of professional information personnel. However, information professionals have limited professional knowledge of nursing. Therefore, training information nurses is undoubtedly a new model of information development under the condition of limited resources [16, 17]. For the large-scale IM software designed in the present study, the system developers themselves were the clinical front-line staff. This saves time for information engineers in collecting key points and the software structure of department instrument use management. After applying the information system, the whole process monitoring of standardization and flow of instrument registration was realized. The results of the present study revealed the following: After the system was used, the relevant information of surgical patients can be automatically collected by the system, and recorded by multiple instruments at the same time, the usage time, cumulative usage time and other items were automatically extracted and calculated, the maintenance information was automatically called, and the one-key sending of maintenance information and other functions were realized, greatly reducing the time for nurses to record, improving work efficiency, and reducing the average recording time by 32 s (*P* < 0.01). The standardization of information system content avoids many disadvantages of written records, and the registration qualified rate increased from 80.93% to 98.00% (*P* < 0.01).

Medical equipment use safety management is a complex MS that consists of medical equipment, the medical environment (software and hardware), users, and clinical medical engineers [18–20]. The information system based on the chain process improves the efficiency of cooperation among different departments. The results of the present study revealed the following: the speed of the cross-departmental information transmission significantly improved, and the awareness rate of the whole staff for equipment repair application and the arrival time of maintenance personnel significantly improved in the observation group (*P* < 0.01). In the present study, multiple information systems were integrated to achieve the construction of a chain process information platform, thereby combining each instrument in the OR into a whole communication and integration information chain. Information system integration takes many forms, including full-time personnel management and data exchange interface design. After its integration, the process achieves the whole life cycle management process of an instrument: the department’s equipment application and demonstration (OA for MS) → material management office's reviews application (OA) → demonstration passes → signing a contract with the winning company → fixed assets administrator (OA) accepts the warehousing information → equipment installation acceptance and debugging → asset warehousing (fixed asset MS) → equipment data maintenance (fixed asset MS) → department collection (fixed asset MS) → department use management (OR-IM system) → equipment maintenance (maintenance management information system) → department submits the equipment scrap application (OA) → the fixed asset administrator files this → asset disposal-scrap management (fixed asset MS) → keeping accounts of the asset (fixed asset MS). The integration system realizes the information maintenance and updates of new equipment in the department from the data interface between the fixed assets MS and the OR-IM system. Then, the data interface design between the OR-IM system and the maintenance management information system realizes the real-time transmission of maintenance information of the instrument and equipment through the department staff. The OA software implements the authority setting and real-time dynamic information reminder for administrators, in order to complete the maintenance of asset receipt, issue, scrap and keeping management information in the fixed asset MS.

## Conclusion

The whole life cycle management of instruments and equipment was realized using the inter-working and integrating information chain management model of large-scale instruments in the OR based on the chain process, and the continuous and effective dynamic monitoring of the use state of instruments and equipment was ensured. This model is not only suitable for the OR, but also for all clinical departments. In future studies, the investigators will continue to improve and standardize the IM library, and explore new models of system operation.

## Data Availability

The datasets used and/or analysed during the current study available from the corresponding author on reasonable request.

## Abbreviation

R&D: Research and development

## References

[1] Lin WH, Zheng FY, Cai DD, Yang Q, Xie LS, Xu M (2016). Applications of “1+3” mode in continuous quality management of OR equipment. Chin J Modern Nurs.

[2] Tao XY (2010). Progressive training method and experience on the use of new nurse equipment in OR. NursRehabil J.

[3] China hospital association patient safety goals (2019 version) [EB/OL]. http://www.cha.org.cn/plus/view.php?aid=15808.

[4] Hu QY, Wu XR, Qin YW, Yang Y, Xi HQ, Qin XN, Zhang YJ (2018). Construction of pressure injury information system for patients with surgery based on chain-type process. Chin J Modern Nurs.

[5] Zhao WQ (2002). Chain type process and chain type management. SciTechnolManag Res.

[6] Ge X (2018). Materials purchasing whole-process management based on hospital multi information platforms. Chin Med Equip J.

[7] Guédon AC, Wauben LS, Overvelde M (2014). Safety status system for operating room devices. Technol Health Care.

[8] Kasparick M, Schmitz M, Andersen B (2018). OR. NET: aservice-oriented architecture for safeand dynamic medical device interoperability. Biomed Tech (Berl).

[9] MDPnP Program. Medical Device “Plug-and-Play”Interoperability Program. 2015-03-14. [Online]. Available: www.mdpnp.org/.

[10] Okamoto, J., Masamune, K., Iseki, H., et al. Developmentof a next-generation operating room “Smart Cyber OperatingTheater (SCOT)”—development concept and project summay. In *Proceedings of CARS 2015: International Conferenceand Exhibition on Computer Assisted Radiology and Surgery, Barcelona, Spain*, 156–158 (2015).

[11] Zhang Y, Wu P (2017). Study on the performance evaluation model of large-scale instruments and equipment in colleges and universities. Med EducManag.

[12] Tang F (2009). Reflections on the management of large equipment in medical colleges and universities. J Military DoctSouthw China.

[13] China hospital association patient safety objectives (The 2019 version) [EB/OL]. http://www.cha.org.cn/plus/view.php?aid=15808.

[14] Shi LP (2009). Clinical application of the American nursing information system. Chin NursManag.

[15] Yang TC, Lai H (2006). Comparison of product bundling strategies on different online shopping behaviors. Electron Commer RA.

[16] Cao YL, An Y (2017). Development and application of reagent warehousing management and tracking system software. Chin J Modern Nurs.

[17] Farnum MA, Mohanty L, Ashok M, Konstant P, Ciervo J, Lobanov VS, Agrafiotis DK (2019). A dimensional warehouse for integrating operational data from clinical trials. Database (Oxford).

[18] Liu YM, Liu GQ, Wang XM (2014). Safety management for clinical application of medical equipment. Chin Med Equip J.

[19] Ishida K, Hirose M, Fujiwara K, Tsuruta H, Ikeda N (2014). Analysis of medical equipment management in relation to the mandatory medical equipment safety manager (MESM) in Japan. J HealthcEng.

[20] Zhang M, Zheng K, Shen Y, Lin Z, Li Z (2018). Hospital networked medical equipment safety management. Zhongguo Yi Liao Qi XieZaZhi.

